# The innate and T-cell mediated immune response during acute and chronic gammaherpesvirus infection

**DOI:** 10.3389/fcimb.2023.1146381

**Published:** 2023-03-31

**Authors:** Viktoria Rex, Razieh Zargari, Markus Stempel, Stephan Halle, Melanie M. Brinkmann

**Affiliations:** ^1^ Institute of Genetics, Technische Universität Braunschweig, Braunschweig, Germany; ^2^ Institute of Immunology, Hannover Medical School, Hannover, Germany; ^3^ Virology and Innate Immunity Research Group, Helmholtz Centre for Infection Research, Braunschweig, Germany; ^4^ Institute of Clinical Chemistry, Hannover Medical School, Hannover, Germany

**Keywords:** EBV, MHV68, gammaherpesvirus, innate immunity, interferon, NK cells, T cells, CTL

## Abstract

Immediately after entry into host cells, viruses are sensed by the innate immune system, leading to the activation of innate antiviral effector mechanisms including the type I interferon (IFN) response and natural killer (NK) cells. This innate immune response helps to shape an effective adaptive T cell immune response mediated by cytotoxic T cells and CD4^+^ T helper cells and is also critical for the maintenance of protective T cells during chronic infection. The human gammaherpesvirus Epstein-Barr virus (EBV) is a highly prevalent lymphotropic oncovirus that establishes chronic lifelong infections in the vast majority of the adult population. Although acute EBV infection is controlled in an immunocompetent host, chronic EBV infection can lead to severe complications in immunosuppressed patients. Given that EBV is strictly host-specific, its murine homolog murid herpesvirus 4 or MHV68 is a widely used model to obtain *in vivo* insights into the interaction between gammaherpesviruses and their host. Despite the fact that EBV and MHV68 have developed strategies to evade the innate and adaptive immune response, innate antiviral effector mechanisms still play a vital role in not only controlling the acute infection but also shaping an efficient long-lasting adaptive immune response. Here, we summarize the current knowledge about the innate immune response mediated by the type I IFN system and NK cells, and the adaptive T cell-mediated response during EBV and MHV68 infection. Investigating the fine-tuned interplay between the innate immune and T cell response will provide valuable insights which may be exploited to design better therapeutic strategies to vanquish chronic herpesviral infection.

## Introduction

### The oncogenic human gammaherpesviruses EBV and KSHV

The *Herpesviridae* are a large family of DNA viruses that infect a wide range of host species. The family comprises three subfamilies, the *Alpha-*, *Beta-*, and *Gammaherpesvirinae*, and they all share the ability to establish chronic, lifelong infections in their hosts. Mostly, herpesviruses cause severe disease only in naïve or immunosuppressed individuals. After primary infection, they establish a state called latency, with the hallmark of minimal viral gene expression and absence of *de novo* synthesis of viral particles (Cohen, 2020). Disruptions can induce reactivation and transition from latency to the lytic replication cycle, which results in viral gene expression and production of new virus progeny and virus dissemination (Davison et al., 2009). Unlike the Alpha- and Betaherpesviruses, the Gammaherpesviruses are oncogenic and are associated with the development of lymphoproliferative diseases and lymphomas as well as multiple other cancers (Wen et al., 2021). The two Gammaherpesviruses known to infect humans are Epstein-Barr virus (EBV or Human Herpesvirus 4) and Kaposi’s sarcoma-associated herpesvirus (KSHV or Human Herpesvirus 8).

KSHV was initially discovered through its tight association with Kaposi’s sarcoma (KS) (Chang et al., 1994; Cesarman et al., 2019) and since then has been associated with a wide spectrum malignancies (Mariggiò et al., 2017). These include B cell lymphoproliferative disorders like primary effusion lymphoma (Nador et al., 1996), multicentric Castleman disease (Bélec et al., 1999; Carbone et al., 2021), diffuse large B cell lymphoma (Dupin et al., 2000), and germinotropic lymphoproliferative disorder (Du et al., 2002). In recent years, further KSHV-associated diseases were added to this list, including KSHV-positive reactive lymphoid hyperplasia and plasmablastic proliferation of the splenic red pulp (Gonzalez-Farre et al., 2017), bone marrow failure in immunosuppressed patients after transplantation (Luppi et al., 2000), and KSHV inflammatory cytokine syndrome (Uldrick et al., 2010). KSHV infection is not ubiquitous, with seroprevalence varying among different populations from high-level endemic areas (mainly occurring in Sub-Saharan Africa with seropositivity rates >50%), intermediate-level endemic areas (Mediterranean countries with seroprevalence rates between 10-30%), and non-endemic areas (most parts of Europe, Asia, and the US with <10%) (Yan et al., 2019). Conversely, EBV infection is ubiquitous, with about 95% of older adults worldwide being infected (Andrei et al., 2019). While EBV is mostly unnoticeably acquired in childhood, it can cause a diverse range of diseases (Damania et al., 2022). For example, EBV is associated with the endemic form of Burkitt’s lymphoma and is involved in the genesis of another geographically restricted cancer, nasopharyngeal carcinoma, as well as a subset of Hodgkin’s lymphoma and gastric carcinoma (Crawford et al., 2014). Chronic EBV infection or reactivation in patients who have been immunocompromised due to organ transplantation can lead to the development of various B cell malignancies known as post-transplant lymphoproliferative disease (PTLD) (Nijland et al., 2016). In some rare cases, individuals are not able to resolve and control EBV infection, which leads to the development of chronic active EBV (CAEBV) disease (Kimura and Cohen, 2017). During CAEBV disease, EBV-positive lymphocytes infiltrate different organs and the viral load in blood is elevated which is often accompanied by fever and enlargement of the spleen (splenomegaly) (Kimura and Cohen, 2017). Very recently, EBV infection has been suggested as a possible cause of multiple sclerosis (MS), a neurodegenerative autoimmune disease, a finding that may open up new directions for clinical trials of MS treatment (Bjornevik et al., 2022).

In the immunocompetent host, EBV and KSHV persist for many years without causing noticeable pathology. However, when the host becomes immunocompromised, long-term persistence *via* latency is postulated to contribute to cancer, and a subset of proteins expressed during the lytic viral life cycle have also been suggested to enhance transformation, possibly *via* auto- and paracrine effects (Manners et al., 2018; Wen et al., 2021).

### The restricted host range of EBV and KSHV requires animal models

The very narrow host range of EBV and KSHV is challenging for studying their pathogenesis, but over the past decades animal models were established that provided important insights into the interaction with the host’s immune system of these oncogenic viruses. Different animal models are required to study EBV and KSHV infection as these viruses are the cause of a diverse set of diseases. To analyze the formation of KS-like tumors, a New-World non-human primate model was established, while humanized mice can be used to simulate PEL-like lymphomas (Fujiwara and Nakamura, 2020), as well as KS (Dubich et al., 2019).

Very few species of laboratory animals can be infected with EBV, including cotton-top tamarins (*Saguinus Oedipus*) and common marmosets (*Callithrix jacchus*). Rabbits inoculated with EBV also exhibit infection, which results in viral DNA load in peripheral blood lymphocytes and serum antibodies specific to EBV. Humanized mice harboring reconstituted human B, T and natural killer (NK) cells, macrophages, and dendritic cells (DCs) after transplantation of human hematopoietic stem cells exhibit infectious mononucleosis-like symptoms, B cell lymphoproliferative disease and latency (Fujiwara, 2018).

As infection of the animals often results in some, but not all, specific disease conditions resembling human EBV- or KSHV-associated disorders, the use of homologous viruses as surrogate models for EBV and KSHV has been explored. Most prominently, the rhesus lymphocryptovirus (rhLCV, Macacine gammaherpesvirus 4), rhesus macaque rhadinovirus (RRV), and murid herpesvirus 4, also known as murine gammaherpesvirus 68 (MHV68) (Fujiwara, 2018; Fujiwara and Nakamura, 2020) have been studied. While rhLCV and RRV infect non-human primates, MHV68 has been widely used as a model virus to study aspects of gammaherpesvirus infection in mice and lies in the focus of this review (Barton et al., 2011; Wang et al., 2021).

Similar to EBV and KSHV, MHV68 infects and exploits B cells to establish latency (Barton et al., 2011; Wang et al., 2021). Mice infected with MHV68 show symptoms similar to infectious mononucleosis including CD8^+^ T cell lymphocytosis and splenomegaly (Tripp et al., 1997; Flaño et al., 2002). Additionally, MHV68 infection can drive tumor development in immunocompromised mice (Barton et al., 2011). Besides, MHV68 is a valuable model to study coinfections with other pathogens such as *Plasmodium falciparum*, helminths or bacteria (Reese, 2016).

### The innate and adaptive immune responses are tightly interlinked

The innate antiviral immune response initiates with the recognition of viruses by specific sensors called pattern recognition receptors (PRRs). Upon activation, induced mostly by virus-derived or aberrantly localized nucleic acids, PRR signaling leads to the initiation of an antiviral inflammatory response (de Oliveira Mann and Hornung, 2021; Guy and Bowie, 2022). This includes secretion of proinflammatory cytokines such as interleukins, tumor necrosis factor (TNF), as well as type I interferons (IFN), resulting in the recruitment of innate immune cells including monocytes, DCs, and NK cells (McNab et al., 2015; Carty et al., 2021). The antiviral innate immune response also initiates and shapes the adaptive immune response with its central players, the cytotoxic CD8^+^ T cells (CTL), CD4^+^ T helper cells, and B cells (Tomalka et al., 2022). CTL can directly attack and kill virus-infected cells, T helper cells secrete soluble factors with antiviral functions, and the antibodies produced by B cells contribute to neutralization of free virus particles.

An inefficient innate immune response to viral infection results in the development of a non-protective adaptive immune response, higher virus replication and antigen burden, which in turn can lead to T cell exhaustion, a mechanism that also might protect the host from severe inflammation (Panetti et al., 2022). Exhaustion of T cells is identified by loss of effector functions including decreased production of IL-2, TNF, and IFNγ cytokines, as well as elevated and persistent expression of inhibitory receptors such as Programmed cell death protein 1 (PD-1). Different factors can lead to T cell exhaustion, with the presence of high antigen burden in addition to long and persistent antigen stimulation being the main contributors during chronic viral infections (McLane et al., 2019).

In this review, we will highlight both the innate immune response, with focus on the type I IFN and NK cell response, and the adaptive immune response mediated by T cells to EBV and its murine homologue MHV68.

## The type I interferon response is crucial to control herpesvirus infections

**Figure 1:**
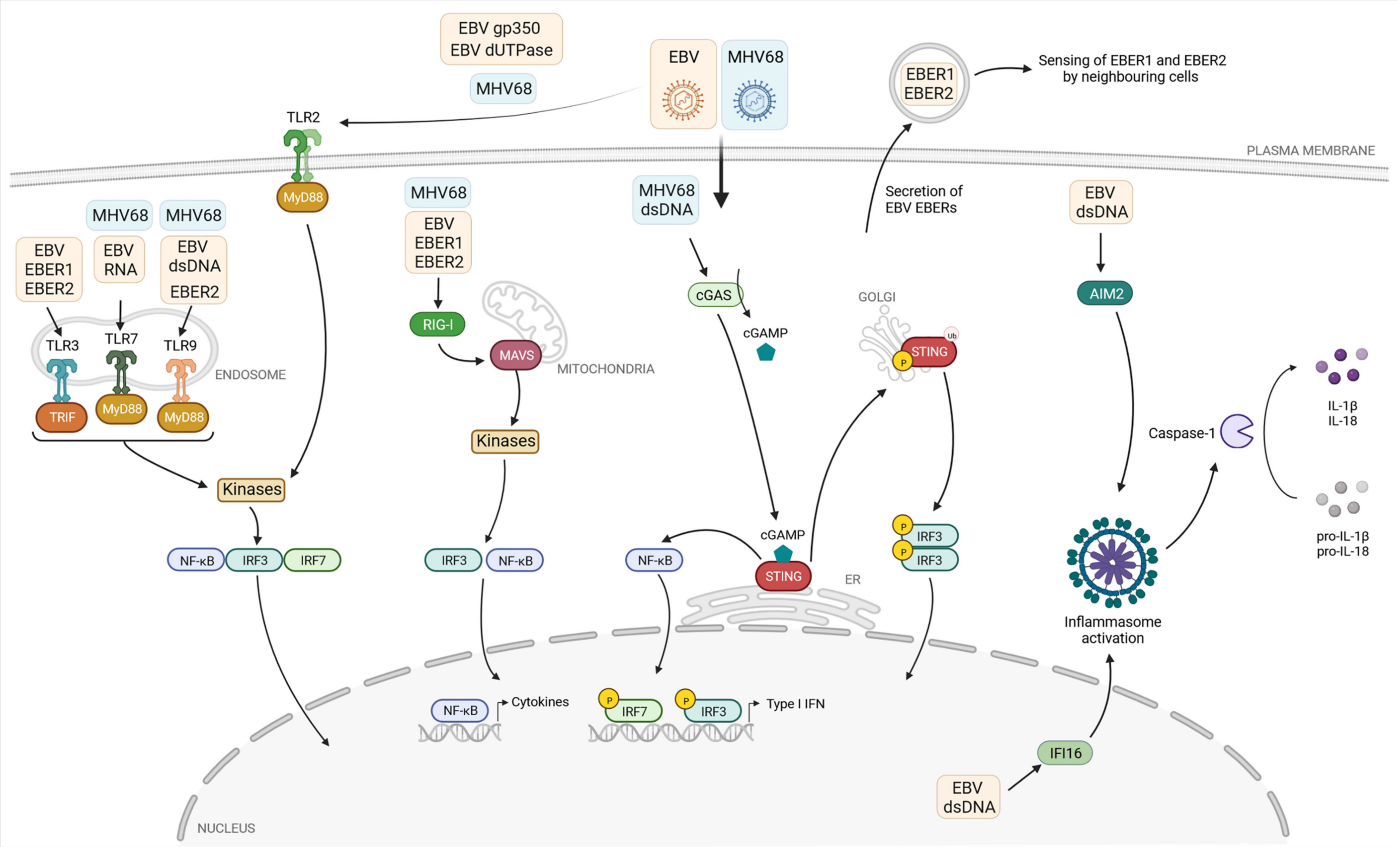
Current knowledge about the detection of the gammaherpesviruses EBV and MHV68 by pattern recognition receptors. The pattern recognition receptors (PRR) Toll-like receptors (TLR) 2, 3, 7, and 9, the cytoplasmic RNA sensor retinoic-acid-inducible protein 1 (RIG-I), and the DNA sensor cyclic GMP-AMP (cGAMP) synthase (cGAS) sense viral proteins or viral nucleic acids associated with EBV (yellow box) or MHV68 (blue box). If known, the viral ligands are depicted in the respective boxes. After binding their ligands, these PRR initiate a signaling cascade, leading to recruitment and/or activation of adaptor molecules: TIR domain-containing adaptor-inducing interferon-β (TRIF) and myeloid differentiation primary-response protein 88 (MyD88) in the case of TLRs, mitochondrial antiviral signaling protein (MAVS) for RIG-I, and stimulator of interferon genes (STING) in the case of cGAS. Subsequently, the transcription factors interferon regulatory factor 3 and 7 (IRF3, IRF7) and nuclear factor-κB (NF-κB) are activated, translocate to the nucleus and induce type I IFN and proinflammatory cytokine expression. The two DNA sensors absent in melanoma 2 (AIM2) and gamma-interferon-inducible protein 16 (IFI16) sense EBV-derived DNA and activate the formation of the inflammasome, leading to caspase-1-dependent formation of the proinflammatory cytokines IL-1β and IL-18. EBER: EBV-encoded small RNA. Created with BioRender.com.

Host cells are equipped with PRRs that are expressed at the cell surface, the cytoplasm, the endolysosomal compartment and the nucleus, where they recognize molecular structures of invading pathogens or sense cellular alterations induced by pathogens (**Figure 1**). Several classes of PRRs are involved in innate immune sensing of gammaherpesviruses, including plasma membrane or endosomal Toll-like receptors (TLRs), cytosolic retinoic acid inducible gene I (RIG-I)-like receptors (RLRs), as well as nuclear and cytosolic DNA sensors, which will be discussed in this review. Upon sensing viral infection, PRRs activate downstream signaling cascades leading to the secretion of type I IFN and proinflammatory cytokines (de Oliveira Mann and Hornung, 2021; Guy and Bowie, 2022). Upon secretion, type I IFN exert their activity in an autocrine and paracrine manner by activation of the type I IFN α/β receptor (IFNAR). Binding of type I IFN to the IFNAR leads to the phosphorylation and activation of the transcription factors signal transducers and activators of transcription 1 and 2 (STAT1 and STAT2), carried out by the IFNAR-associated kinases tyrosine kinase 2 (TYK2) (Yan et al., 1996) and Janus kinase 1 (JAK1) (Domanski et al., 1997). Activated STAT1 and STAT2 form a trimeric complex with interferon regulatory factor 9 (IRF9) and translocate into the nucleus, resulting in the induction of interferon-stimulated gene (ISG) expression, whose products mediate broad antiviral activities (Stark and Darnell, 2012).

The type I IFN response plays a vital role not only for protecting single cells from viral infection, but also for initiating inflammation and shaping an effective innate and adaptive immune response (Le Bon and Tough, 2002; Ivashkiv and Donlin, 2014). Therefore, type I IFN deficiencies, including non-functional PRR or IFNAR signaling, are usually detrimental and lead to fatalities due to massive viral spread and the inability of the host immune system to control the acute infection as revealed by *in vivo* studies in mice (Meyts and Casanova, 2021). In humans, such deficiencies or inborn errors in the type I IFN response or its induction lead to complications with different severities, following vaccination with live-attenuated viruses, or infection with herpesviruses and respiratory viruses such as SARS-Coronavirus 2 and Influenza Virus (Casrouge et al., 2006; Hernandez et al., 2019; Bastard et al., 2020; Carter-Timofte et al., 2020; Zhang et al., 2020; Bastard et al., 2021; Andreakos et al., 2022; Gothe et al., 2022; Manry et al., 2022; Mogensen, 2022; Zhang et al., 2022b; Zhang et al., 2022a). To date, the knowledge about type I IFN deficiencies and the outcome of infections with human gammaherpesvirus is limited, probably due to their rare appearance in human populations or due to the absence of clinical complications following most infections (Meyts and Casanova, 2021).

While *in vivo* evidence for EBV and KSHV control by the type I IFN system is sparse, the MHV68 mouse model provides important insights into its crucial role. Although MHV68 antagonizes the type I IFN response in various ways, the type I IFN response still remains crucial for controlling acute MHV68 infection, latency and reactivation in mice (Wang et al., 2021; Schwerk et al., 2022). The transcription factor interferon regulatory factor 3 (IRF3), which is activated by PRRs, has been shown to be responsible for the induction of type I IFN following MHV68 lytic infection in primary macrophages in an IFNAR-dependent manner (Wood et al., 2013). While WT mice efficiently control acute infection, *Ifnar1* ^-/-^ mice are highly susceptible depending on the viral dose. While 80-90% of *Ifnar1* ^-/-^ mice succumb to MHV68 infection following a high dose intranasal infection (4x10^6^ PFU), ~50% survive low dose infection (4x10^3^ PFU), with viral titers in the lungs being 100-1000 fold higher in KO mice at both high and low doses. In addition, MHV68 disseminates systemically faster in *Ifnar1* ^-/-^ mice (Dutia et al., 1999). Interestingly, genetically modified MHV68 recombinant viruses producing mIFNα1 are attenuated *in vivo* while they can still establish latency in the spleen (Lenschow et al., 2007; Aricò et al., 2011).

### Toll-like receptors: Sensing viral infection at the plasma membrane and in endolysosomes

To date, 10 human (TLR1-TLR10) and 12 murine members (TLR1-TLR9 and TLR11-TLR13) of the TLR family have been identified (El-Zayat et al., 2019; Duan et al., 2022). TLRs are composed of an extracellular domain in charge of sensing pathogen-associated molecular patterns (PAMPs), one transmembrane domain and a cytoplasmic C-terminal domain which mediates the signaling activity *via* cytoplasmic adapter proteins. TLRs localize to the cell surface or within the intracellular endolysosomal compartment. Cell-surface TLRs include TLR1, TLR2, TLR4, TLR5, TLR6, and TLR10, while intracellular TLRs comprise TLR3, TLR7, TLR8, TLR9, TLR11, TLR12, and TLR13 (Kawasaki and Kawai, 2014). Upon binding their specific ligand, conformational changes of the transmembrane receptors lead to the recruitment and binding of the adaptor molecules myeloid differentiation primary-response protein 88 (MyD88), MyD88-adaptor-like protein (MAL) and TIR domain-containing adaptor-inducing interferon-β (TRIF), with MyD88 being the key adaptor for most TLRs. Upon activation, MyD88 recruits kinases to induce a complex signaling response, eventually leading to the phosphorylation and activation of transcription factors including nuclear factor-κB (NF-κB), IRF3, and IRF7. TLR3 does not recruit MyD88, but instead recruits TRIF to drive an individual signaling pathway, resulting in the similar activation of NF-κB signaling (**Figure 1**) (Sartorius et al., 2021; Duan et al., 2022).

TLR2, TLR3, TLR7, and TLR9 have been described to contribute to the detection of gammaherpesviruses (**Table 1**). Upon infection of TLR2-transfected HEK293 cells, UV-inactivated EBV particles strongly induce NF-κB activation and secretion of the chemokine monocyte chemoattractant protein-1 (Gaudreault et al., 2007). Furthermore, by pre-treating TLR2-expressing cells with the viral DNA polymerase inhibitor phosphonoacetic acid and infection with UV-inactivated EBV, NF-κB levels were also increased, indicating that recognition by TLR2 is not dependent on viral replication, but likely induced by binding of EBV particles on the cell surface. Further experiments suggested that TLR2 may recognize the viral surface glycoprotein gp350 (**Figure 1**), which mediates viral entry into the host cell. Another study based on HEK293 cells identified the EBV-encoded deoxyuridine triphosphate nucleotidohydrolase (dUTPase) as a PAMP, also thought to be recognized by TLR2 (**Figure 1**) (Ariza et al., 2009). However, further investigations are needed to verify the reported connection between TLR2 and EBV-encoded dUTPase, starting with the examination of possible dUTPase release to the extracellular milieu from EBV infected cells. For MHV68, NF-κB activation in TLR2-transfected HEK293 cells was shown and experiments in primary mouse embryonic fibroblasts (MEF) from WT and TLR2 KO mice showed a TLR2-dependent IL-6 and IFNα response (**Figure 1**) (Michaud et al., 2010a). Further, MHV68-infected TLR2-deficient mice show decreased IFNα level in their lungs after intranasal infection and increased viral titers. However, upon intravenous infection we could not find evidence for a role of TLR2 during acute MHV68 infection (Bussey et al., 2019). To date, TLR2 is the only cell surface TLR proposed to recognize EBV and MHV68, but the precise nature of the viral ligands was not shown convincingly yet, and its role during EBV infection of its human host is not known.

**Table 1:** Activated innate immunity pathways after EBV and MHV68 infection.

PAMP	PRR	Outcome	Reference
EBV			
EBV particles, probably gp350	TLR2	NF-κB activation and secretion of chemokine monocyte chemoattractant protein-1	Gaudreault et al. (2007)
EBV-encoded dUTPase	TLR2	Activation of NF-κB and secretion of IL-6	Ariza et al. (2009)
EBERs	TLR3	Secretion of TNFα and IL-6	Li et al. (2015)
EBER1	TLR3	Secretion of IFNβ	Iwakiri et al. (2009)
EBV M81-encoded small RNA 2	TLR7	Expression of CXCL8	Li et al. (2019)
Unknown	TLR7 and TLR9	Secretion of IFNα	Fiola et al. (2010)
EBV DNA and RNA	TLR7 and TLR9	Secretion of IFNα	Quan et al. (2010)
Purified EBV DNA	TLR9	Secretion of IL-8	Fiola et al. (2010)
Unknown	TLR9	Activation of T cells	Lim et al. (2007)
EBERs	RIG-I	Production of IFNβ	Ablasser et al. (2009)
EBER1	RIG-I	NF-κB activation and induction of IFNβ	Baglio et al. (2016)
EBERs	RIG-I	Type I IFN response	Samanta et al. (2006)
Unknown	RIG-I	Upregulation of type I IFN and induction of IL-10	Samanta et al. (2008)
EBERs	RIG-I	Upregulation of RIG-I expression	Duan et al. (2015)
Unknown	RIG-I	Secretion of TNFα, IL-6 and IL8	Chiang et al. (2018)
EBER1	RIG-I	Production of TNFα and IL-6	Burassakarn et al. (2021)
Unknown	Inflammasome	Elevated IL-18	Setsuda et al. (1999) and van de Veerdonk et al. (2012)
Unknown	Inflammasome	Elevated IL-1β	Foss et al. (1994)
EBV dsDNA	IFI16	Inflammasome assembly, activation of caspase-1 and secretion of IL-1β	Ansari et al. (2013) and Dutta et al. (2015)
EBV genome	IFI16	Activation of caspase-1 and secretion of IL-1β, IL-18 and IL-33	Ansari et al., 2013; Ansari et al., 2015
EBV genome	AIM2	Inflammasome activation and secretion of IL-1β	Torii et al. (2017)
MHV68			
Unknown	TLR2	NF-κB activation	Michaud et al. (2010a)
Unknown	TLR2	Secretion of IL-6 and IFNα	Michaud et al. (2010a)
Unknown	TLR2	Secretion of IFNα	Michaud et al. (2010a)
Unknown	TLR7 and TLR9	Secretion of IFNα	Bussey et al. (2019)
Unknown	TLR9	secretion of IFNα, IL-6 and IL-12	Guggemoos et al. (2008)
Unknown	TLR9	Production of type I IFN	Luckhardt et al. (2011)
Accumulated host non-coding RNAs	RIG-I	Activation of NF-κB	Karijolich et al. (2015)
MHV68 DNA	cGAS/STING	Secretion of IFNβ	Yang et al. (2015)
Unknown	STING	Induction of necroptosis in a TNF-dependent manner	Schock et al. (2017)

PAMP, pathogen-associated molecular pattern; PRR, pattern recognition receptor; EBER, EBV-encoded small RNA.

The endosomal TLRs TLR3, TLR7, and TLR9 are responsible for detection of viral nucleic acids. While double-stranded RNA (dsRNA) is recognized by TLR3, TLR7 senses fragments of single-stranded RNA (ssRNA) and TLR9 preferentially recognizes ssDNA containing unmethylated CpG motifs (Alexopoulou et al., 2001; Bauer et al., 2001; Heil et al., 2004). The recognition of gammaherpesviral dsRNA by TLR3 will be illuminated in the RNA-sensing section.

During EBV infection, TLR7 and TLR9 were shown to sense EBV nucleic acids and induce an antiviral response in primary monocytes, plasmacytoid dendritic cells (pDC), and B cells (**Figure 1**) (Lim et al., 2007; Fiola et al., 2010; Quan et al., 2010; Li et al., 2019). By applying an inhibitor of endosomal TLR activation, TLR9 is shown to recognize purified EBV DNA in primary monocytes, resulting in IL-8 secretion. pDC stimulated with EBV secrete IFNα, which can be reduced by adding specific inhibitors of TLR9 or, to a lesser extent, TLR7 (Fiola et al., 2010). Besides, TLR9- and TLR7-dependent IFNα production induced by EBV DNA and RNA, respectively, was demonstrated in pDC (Quan et al., 2010). In humanized mice, it was shown that EBV-stimulated pDC contribute to the activation of T cells in a TLR9-dependent manner (Lim et al., 2007). A recent study showed a strain-specific effect of EBV strain M81, which was originally isolated from a nasopharyngeal carcinoma. EBV M81-encoded small RNA 2 (EBER2) increases TLR7-dependent expression of the chemokine CXCL8, leading to spontaneous lytic replication in infected B cells, which is not observed with EBERs transcribed from the EBV B95-8 or Akata strains (Li et al., 2019). This suggests that strain-specific polymorphisms may results in different outcomes of the antiviral immune response, enhancing the complexity of studying virus-host interactions.

Consistent with the findings in human cells, TLR9 is also involved in the detection of MHV68 in murine DCs, being responsible for IFNα, IL-6, and IL-12 secretion (**Figure 1**) (Guggemoos et al., 2008). Moreover, the authors observed an increased viral load in the spleen of TLR9-depleted mice after intraperitoneal infection. In another model, TLR9 expression is shown to be involved in the protection from MHV68-induced lung fibrosis, and required for type I IFN production in the lungs of intranasally infected animals (Luckhardt et al., 2011). Our own work confirmed the important role of TLR9, but also highlighted that TLR7 contributes to the IFNα response of pDC to MHV68 infection (**Figure 1**). While IFNα secretion is reduced in *Tlr9* ^-/-^ cells compared to WT cells, it is only completey abolished in *Tlr7* ^-/-^ *Tlr9* ^-/-^ double-knockout pDC (Bussey et al., 2019). Thus, the only PRRs contributing to the IFN-α response to MHV68 in pDC are TLR7 and TLR9, but the contribution of TLR7 is masked by the presence of TLR9. Congruently, lytic replication of MHV68 after intravenous infection is enhanced in the liver and spleen of *Tlr7* ^-/-^ *Tlr9* ^-/-^ mice. In addition, latent viral loads and reactivation of MHV68 are enhanced in latently infected *Tlr7* ^-/-^ *Tlr9* ^-/-^ splenocytes (Bussey et al., 2019).

In summary, TLR7 and TLR9 both contribute to detection and control of MHV68 infection *in vivo*. The exact nature of the TLR7 and TLR9 ligands during MHV68 infection has not been shown yet. For EBV, the data on TLR2, TLR7 and TLR9 are scarce but can now be generated with the tools of the Cas9 and genomics era, at least *in vitro*.

### How RNA sensors detect DNA viruses: TLR3 and the cytoplasmic sensor RIG-I

At first sight it seems counterintuitive that DNA viruses, which replicate their DNA genome in the nucleus and transcribe their genes with the cellular nuclear transcription machinery, are sensed by cytosolic or endosomal RNA sensors. However, several studies suggest that cellular sensors of dsRNA, namely TLR3 and RIG-I, indeed play a role for detection of gammaherpesviruses (**Table 1**).

EBV encodes non-coding and non-polyadenylated RNAs called EBERs 1 and 2. These small RNAs can adopt secondary structures containing multiple intramolecular stem-loops which resemble dsRNA structures (Rosa et al., 1981; Glickman et al., 1988) and are transcribed by the host DNA-dependent RNA polymerase III from the EBV genome (Rosa et al., 1981; Arrand and Rymo, 1982). TLR3 senses dsRNA in endosomes, and two studies have shown that it seems to detect EBERs (**Figure 1**) (Iwakiri et al., 2009; Li et al., 2015). One of them shows that exogenously expressed EBERs induce inflammatory responses through TLR3 in nasopharyngeal carcinoma cells (Li et al., 2015). The other study addresses the possible underlying mechanism of TLR3-sensing of EBERs: EBER1 binds the cellular lupus erythematosus-associated antigen (La) to evade degradation, and this interaction induces the active secretion of EBER1-La complexes with the possibility of being secreted as an exosome during EBV infection (**Figure 1**). Upon endocytosis of EBER1-La-containing exosomes, TLR3 can recognize EBER1 and induce downstream signaling (Iwakiri et al., 2009). However, further studies are needed to clarify how the EBER1-La complex is recognized by TLR3 and the exact mechanism of EBER1 release.

The cytoplasmic RNA helicases retinoic-acid-inducible protein 1 (RIG-I, also known as Ddx58) and melanoma differentiation-associated protein 5 (MDA5) belong to the RIG-I-like receptor (RLR) family. While RIG-I preferentially binds short, 5’ di- and triphosphorylated ssRNAs, as well as dsRNA, MDA5 preferentially recognizes long dsRNA in the cytoplasm (Hornung et al., 2006; Wang et al., 2010; Peisley et al., 2011). Binding of RIG-I or MDA5 to their respective ligands results in the activation of the mitochondrial antiviral signaling protein (MAVS), which is associated with mitochondria. Activated MAVS induces downstream signaling of TBK1/IRF3 or IKK/NF-κB leading to the production of type I IFN and proinflammatory cytokines, respectively. Several studies highlight the role of EBV-encoded EBER transcripts, which are sensed by RIG-I (**Figure 1**).

RNA polymerase III, responsible for producing the EBER transcripts as well as other cellular small RNAs such as rRNAs and tRNAs, was shown to induce RIG-I-dependent IFNβ production in EBV-infected cells (Ablasser et al., 2009). It does so by converting cytosolic poly(dA-dT) DNA into the RIG-I ligand 5’-phosphorylated dsRNA (Ablasser et al., 2009; Chiu et al., 2009). Since inhibition of RNA polymerase III leads to suppressed EBER1/2 RNA transcription, resulting in a lower RIG-I-dependent activation of IFNβ production (Ablasser et al., 2009). RNA polymerase III seems to contribute to the detection of EBV infection by transcribing EBV-derived small RNAs. This is also supported by the finding that EBER1 can be transferred *via* exosomes to uninfected DCs and trigger antiviral immunity in a RIG-I-dependent manner (Baglio et al., 2016).

Additional studies highlight RIG-I as a critical sensor for EBER1 and EBER2. The direct interaction of RIG-I and both EBERs was shown after transfection of RIG-I-expressing plasmids into EBER-positive EBV-infected cells followed by RIG-I immunoprecipitation and reverse-transcription PCR (RT-PCR) for EBERs (Samanta et al., 2006). Furthermore, a RIG-I-dependent type I IFN response can be detected after reintroducing EBERs in EBER-knockout EBV. Besides the RIG-I-dependent upregulation of type I IFN upon EBV infection in EBV-infected cancer cells, it is demonstrated that IL-10 is induced by RIG-I. Knockdown of RIG-I downregulated IL-10 secretion in EBER-positive EBV-infected cells, which is dependent on the transcription factor IRF3 (Samanta et al., 2008). Furthermore, it is shown that EBV activates RIG-I by disrupting binding of nuclear 5S rRNA pseudogene transcripts with binding partners leading to their unshielding, and thereby recognition by RIG-I, but not MDA5 (**Figure 1**) (Chiang et al., 2018). While the exact mechanism driving the re-localization of host nuclear 5S rRNA is unknown, it was hypothesized that the EBV-mediated host shut-off of the cellular translation machinery plays an important role in inhibiting expression of host proteins which regulate the localization of the nuclear RNA pseudogene transcripts. The link between transcription of EBERs and RIG-I is furthermore shown in the nasopharyngeal carcinoma-derived HNE2 cell line transfected with EBERs which resulted in upregulation of RIG-I expression in a dose-dependent manner (Duan et al., 2015). Aside from IL-10 and type I IFN, TNFα, IL-6, and IL-8 transcripts are also upregulated in EBV-reactivated gastric adenocarcinoma cells in a RIG-I-dependent manner (Chiang et al., 2018).

While human B lymphocytes and epithelial cells are the major targets of lytic and latent EBV infection, several studies have demonstrated the involvement of monocytes in EBV infection (Savard et al., 2000; Masy et al., 2002; Michaud et al., 2010b). One mechanism showing the interplay between EBV and the innate immune response of human monocyte-derived macrophages (MDM) includes incoming exosomes containing EBER1 transcripts, which induce TNFα and IL-6 production in a RIG-I-dependent manner, promoting indoleamine 2, 3-diocygenase (IDO) expression in the cells. Activation of IDO creates an immunosuppressive microenvironment, which negatively affects T cell responses by suppressing the proliferation and cytolytic activity of CD4^+^ and CD8^+^ T cells (Burassakarn et al., 2021), suggesting a possible role of EBERs for inhibition of the adaptive immune response.

In summary, it is not clear how exactly EBERs are secreted into the supernatant of infected cells, either by active secretion in complex with a host protein or in form of extracellular vesicles such as exosomes or microvesicles (Zhao et al., 2019). To date, the properties of these EBER-containing vesicles and their specific roles in the viral life cycle remain largely unclear and the exact mechanism of innate immune detection needs further validation.

Similar to EBV, the involvement of RNA polymerase III was reported in the context of MHV68 infection. RNA polymerase III-dependent transcription of host non-coding RNAs, such as nucleolar protein 14 and Go-Ichi-Ni-San complex subunit 1, can be sensed by RIG-I and activate NF-κB. Infection with MHV68 results in an accumulation of these stimulatory host RNAs, showing an indirect mechanism for RIG-I-dependent sensing of gammaherpesvirus infection (**Figure 1**) (Karijolich et al., 2015). In line with this, MEF lacking functional RIG-I are found to be more permissive to MHV68 infection compared to cells expressing functional RIG-I (Inn et al., 2011).

Taken together, these data point to a critical role of the RNA sensors TLR3 and RIG-I to detect human and murine gammaherpesvirus infection through the recognition of stimulatory virus- as well as host-derived non-coding RNAs.

### DNA sensors: Potent activators of the antiviral response

Intracellular DNA is recognized by different sensors, including the proteins type I IFN inducible protein absent in melanoma 2 (AIM2) (Hornung et al., 2009), gamma-interferon-inducible protein 16 (IFI16) (Unterholzner et al., 2010), and cyclic GMP-AMP synthase (cGAS) (Wu et al., 2013) (**Table 1**). Upon binding to dsDNA in a length-dependent manner, cGAS catalyzes the formation of the second messenger 2’3’-cGAMP which binds to the endoplasmic reticulum (ER)-resident adaptor protein stimulator of IFN genes (STING) leading to its activation (Liu et al., 2015). Originally, cGAS was identified as a cytosolic sensor, but recent studies indicate that cGAS also resides in the nucleus (Volkman et al., 2019; Pathare et al., 2020; Sun et al., 2021). Upon activation, STING translocates from the ER to the Golgi apparatus (Ishikawa and Barber, 2011). Located at the Golgi, STING undergoes several poly-ubiquitinations (Tsuchida et al., 2010; Zhang et al., 2012; Wang et al., 2014), which lead to the phosphorylation of STING followed by the phosphorylation and activation of IRF3 and subsequent type I IFN expression (Liu et al., 2015). Moreover, upon activation, ER-resident STING activates NF-κB prior to its translocation to the Golgi apparatus, mediating the expression of proinflammatory cytokines (Stempel et al., 2019b).

While many studies highlight cGAS/STING signaling during herpesvirus infection and herpesviral evasion of this pathway (Stempel et al., 2019a), research into the role of DNA sensors in the context of EBV infection is sparse. For EBV, most studies are restricted to B cells, as the virus exploits B cells as a reservoir for persistent infection. Interestingly, uninfected B cells were found to lack detectable STING expression, while EBV-infected cells did express cGAS and STING, yet were not able to produce type I IFN upon dsDNA stimulation (Gram et al., 2017). Correlating with this observation, EBV has been shown to induce the E3 ligase TRIM29 in epithelial cells, which regulates K48-linked ubiquitination and degradation of STING, preventing the activation of cGAS-STING signaling (Xing et al., 2017). However, if this scenario contributes to the dysfunction of cGAS-STING signaling in EBV-infected B cells or if EBV inhibits cGAS/STING activation by a hitherto unrecognized mechanism warrants further investigation.

In contrast to EBV, the cGAS/STING pathway was found to be relevant during MHV68 infection (**Figure 1**). Upon intraperitoneal infection, viral titers are increased in the spleens and lungs of cGAS-deficient mice, confirming the importance of this signaling pathway for the antiviral immune response (Schoggins et al., 2014). Following stimulation with MHV68 DNA, cGAS/STING-dependent signaling is activated in mesenchymal stem cells resulting in IFNβ secretion (Yang et al., 2015). Furthermore, MHV68 infection is capable of inducing necroptosis in a murine fibrosarcoma cell line through STING in a TNF-dependent manner (Schock et al., 2017), proposing distinct roles of STING in the response to gammaherpesvirus infection in mice.

### Inflammasomes: Multiprotein complexes engaging in cytokine secretion

Inflammasomes are intracellular multiprotein complexes that are assembled upon pathogen recognition or danger signals. Members of the NOD-like-receptor (NLR) family, i.e. NLRP1, NLPR3, or NLRC4, are associated with inflammasome formation. AIM2 is another cytoplasmic sensor for inflammasome activation that recognizes dsDNA (Hornung et al., 2009; Hu et al., 2016). Different herpesviruses activate distinct inflammasome-activating sensors, e.g. HSV-1 was demonstrated to activate the NLRP3 inflammasome (Karaba et al., 2020), HCMV activates the AIM2 inflammasome (Botto et al., 2019), while KSHV is shown to induce IFI16-dependent inflammasome activation (Kerur et al., 2011). For EBV and MHV68, multiple inflammasome-activating pathways were identified, which will be discussed in this section (**Table 1**).

Canonical inflammasomes are composed of three major components: a sensor protein, a complex called adaptor-apoptosis-associated-speck-like-protein-containing-a-caspase-recruitment domain (ASC) and caspase-1 (**Figure 1**) (Evavold and Kagan, 2019). Upon activation, the sensor protein recruits ASC molecules which undergo oligomerization, followed by the recruitment of pro-caspase-1. Pro-caspase-1 catalyzes autolysis to produce the active caspase-1, followed by caspase-dependent cleavage of immature forms of the proinflammatory cytokines IL-1β and IL-18. Mainly myeloid cells such as macrophages produce IL-1β to mediate immune responses against pathogens and tissue damage (Hornung et al., 2009). While IL-1β is shown to be a potent proinflammatory cytokine that is crucial for host-defense to infection and injury, but also for the polarization of CD4^+^ T cells and the activation and differentiation of antigen-specific CTL (Dinarello, 1996; Nambu et al., 2006; Ben-Sasson et al., 2013; Garlanda et al., 2013), IL-18 is demonstrated to induce the differentiation of CD4^+^ to T helper cells 1 (Th1) and Th2, regulating their immune responses, and to drive NK cell and CTL activity through the promotion of IFNγ production (Xu et al., 2000; Nakanishi et al., 2001; Dupaul-Chicoine et al., 2015).

Previous studies observed elevated IL-18 levels in EBV-induced infectious mononucleosis, indicating the activation of the inflammasome (Setsuda et al., 1999; van de Veerdonk et al., 2012). Similarly, IL-1β is found to be elevated in the tonsils of children infected with EBV (Foss et al., 1994). In infected B cells, IFI16-dependent and AIM2-independent inflammasome assembly, production of active caspase-1, and IL-1β secretion is shown, which relies on episomal dsDNA binding of IFI16 in the nucleus (**Figure 1**) (Ansari et al., 2013; Dutta et al., 2015). The colocalization of IFI16 and the EBV genome is demonstrated by immunofluorescence of latently infected B cells and this colocalization results in the acetylation of IFI16 and association with ASC in *de novo* infected primary B cells, followed by cleavage of pro-caspase-1 and secretion of IL-1β, IL-18, and IL-33 (Ansari et al., 2013; Ansari et al., 2015). Moreover, Epstein-Barr nuclear antigen 1 (EBNA1) and EBERs could be excluded from being responsible for inflammasome activation. Interestingly, the knockdown of endogenous IFI16 results in enhanced levels of EBV lytic gene expression as well as an increase in EBV genome abundance, while overexpression of IFI16 reverses these effects (Pisano et al., 2017; Torii et al., 2017), suggesting that IFI16 is critical for controlling EBV replication and gene expression.

Apart from nuclear IFI16, the cytoplasmic dsDNA sensor AIM2 is involved in EBV genome sensing and inflammasome activation (**Figure 1**). In the human monocytic cell line THP-1 and primary human monocytes, EBV infection leads to the release of IL-1β. AIM2 expression is upregulated in infected cells, and knockdown of AIM2 attenuates IL-1β release (Torii et al., 2017), indicating that AIM2-dependent activation of the inflammasome is triggered by EBV infection.

In summary, multiple inflammasome pathways play a role during EBV infection, but the cell type-specificity of inflammasome activation and its effects on the adaptive immune response during the different stages of EBV infection have yet to be analyzed.

### Too strong to be tolerated: EBV and MHV68 evade the PRR-mediated type I IFN response

The fact that herpesviruses evolved multiple strategies to modulate their host’s immune response clearly highlights the power of the antiviral response to control infection. Studies analyzing the role and mechanism of virally encoded immune evasions clearly substantiated our knowledge about the essential role of the type I IFN response to control viral infection. MHV68 evolved different strategies to evade the type I IFN response to promote the primary lytic infection, obvious by hardly detectable type I IFN secretion in *in vitro* infected cells (Bussey et al., 2018). Herpesviral immune evasion appears at multiple levels of the innate immune response. To avoid the DNA-sensing pathway after entry into the host cell, MHV68-encoded ORF64 efficiently delivers viral DNA to the nucleus, while loss of ORF64 results in accumulated localization of viral DNA in the cytoplasm, leading to the activation of STING and AIM2 (Sun et al., 2015). Furthermore, MHV68-encoded ORF11, a virion-associated tegument protein, binds to the kinase TBK1 and disrupts its interaction with IRF3, thereby inhibiting IRF3-mediated induction of *ifnb1* transcription (Kang et al., 2014). MHV68 ORF36, a conserved herpesviral kinase, inhibits IFNβ production by interacting with the activated form of IRF3 inside the nucleus, thereby suppressing the recruitment of RNA polymerase II to the IFNβ gene promoter. The lack of ORF36 leads to the attenuation of the virus *in vitro* and *in vivo*, causing delayed but not completely impaired establishment of latency (Hwang et al., 2009). Moreover, the MHV68 M2 latency protein contributes to inhibition of the type I IFN response by downregulating STAT1 and STAT2 expression in fibroblasts and B lymphocytes (Liang et al., 2004). The EBV-encoded microRNA *Bam*HI fragment A rightward transcript 16 (BART16) suppresses type I IFN signaling *via* directly targeting Cyclic adenosine monophosphate Response Element Binding protein (CREB), which is a key transcriptional activator of the type I IFN signaling pathway (Hooykaas et al., 2017). Additionally, the EBV early protein nuclear egress protein 2 suppresses IFNβ transcription by inhibiting IRF3 activation (Wang et al., 2020). The EBV tegument protein BGLF2 suppresses type I IFN signaling by binding to Tyk2 and suppressing JAK-STAT signaling through recruitment of Src homology region 2 domain-containing phosphatase-1 (SHP1) phosphatase, promoting STAT2 degradation, which leads to decreased expression of ISGs including IRF1, IRF7, and MxA (Liu et al., 2020; Jangra et al., 2021). This large portfolio of herpesviral immune modulators highlights the necessity to dampen the innate immune response at multiple levels and allow the virus to gain a foothold in its host.

### The role of the type I IFN response in shaping the immune response to gammaherpesvirus infection

**Figure 2:**
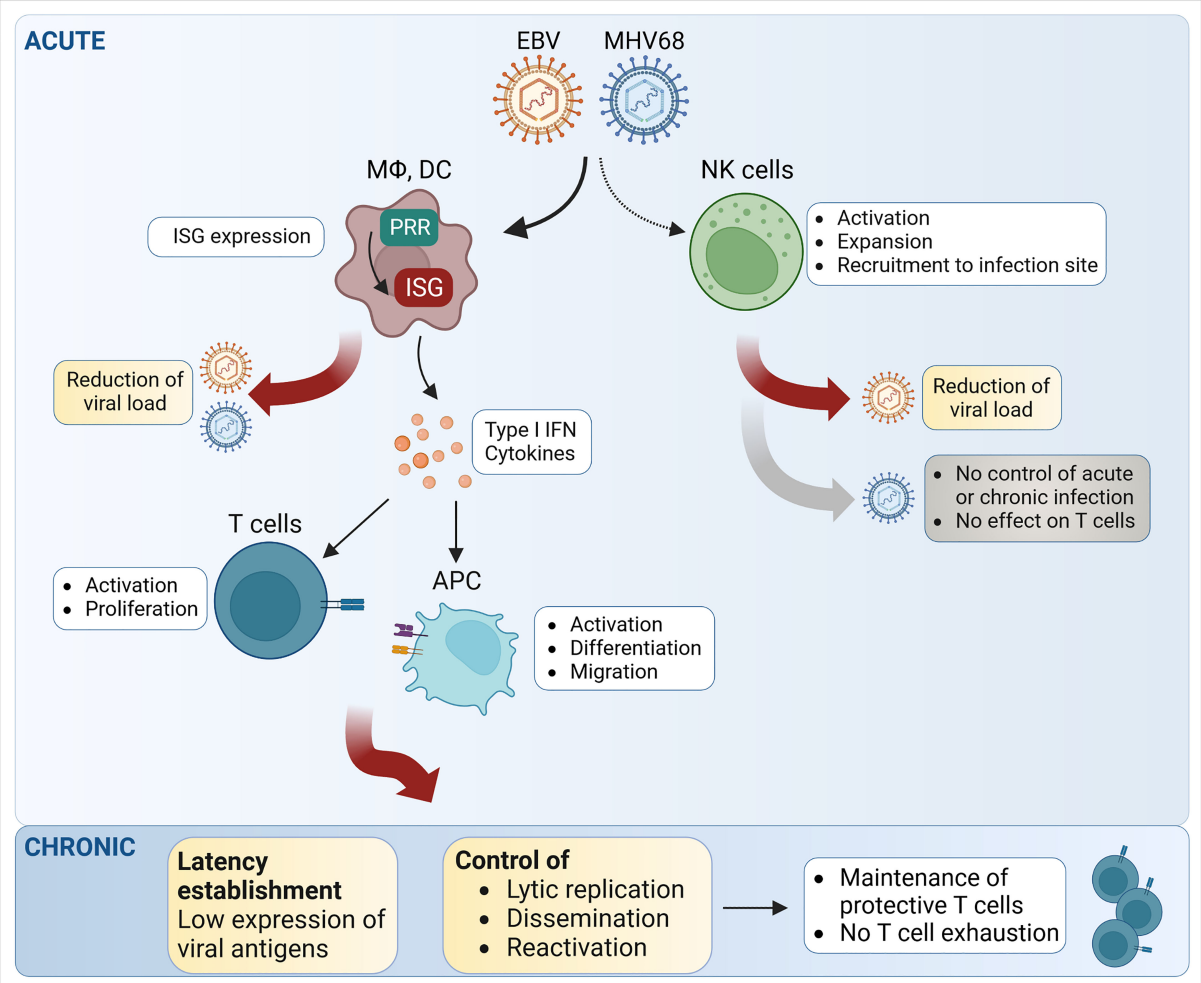
Schematic representation of the main steps of NK- and T cell-mediated control of EBV and MHV68 infection. During acute infection, both EBV and MHV68 trigger natural killer (NK) cell activation, recruitment and expansion. However, the relative impact on the control of MHV68 infection and MHV68-triggered T cell responses appears limited, while NK cells seem to have an effect on EBV replication. Upon detection of EBV and MHV68, pattern recognition receptors (PRR) induce the expression of interferon-stimulated genes (ISG), which exert direct effects on critical steps of the viral life cycle. By inducing type I IFN and proinflammatory cytokine secretion, EBV and MHV68 can trigger important downstream effects leading to T cell activation and T cell proliferation. During chronic infection, the type I IFN response and T cells both contribute to the control of latency and reactivation. APC, antigen-presenting cells; DC, dendritic cells; MΦ, macrophages. Created with BioRender.com.

Type I IFN and proinflammatory cytokines are critical for the maturation of other innate and adaptive immune cells, such as NK and cytotoxic T cells, and guide their recruitment to the site of infection, where they contribute to recognition of virus infection, cytokine production, and killing of infected cells (Wedekind et al., 2020). In addition to directly impeding viral replication, type I IFN also helps to shape an effective adaptive immune response (**Figure 2**), however, this appears to be context-dependent and complex.

Type I IFN can impact the priming of adaptive cell-mediated immune responses in many ways such as by promoting differentiation of myeloid precursors into DCs (Santini et al., 2000; Dauer et al., 2003), upregulating the expression of major histocompatibility complex (MHC) and co-stimulatory molecules such as CD80 and CD86 (Montoya et al., 2002), and promoting the migration of DCs from tissues to lymph nodes by upregulation of chemokine receptor 7 expression, which ultimately promotes antigen presentation and priming of T cells in the lymph nodes (Parlato et al., 2001; Rouzaut et al., 2010). In addition to indirectly affecting T cell priming and activation, the type I IFN response can directly promote or inhibit T cell proliferation and cytokine production depending on the activation status of T cells and their antigen specificity (Marshall et al., 2011; Keppler et al., 2012; Welsh et al., 2012).

The impact of the type I IFN response on gammaherpesvirus infection and the developing adaptive immune response has been more intensively studied for MHV68 than for EBV. The type I IFN response is essential for the control of acute and latent MHV68 infection (Mboko et al., 2017; Schwerk et al., 2022). Following MHV68 infection of *Ifnar1* ^-/-^ mice, the expression of TNF-α, IFNγ, and IL-2 is decreased in antigen-specific CD8^+^ T cells, showing clear hallmarks of T cell exhaustion in the absence of type I IFN signaling (Jennings et al., 2014). Thus, intact type I IFN signaling is important for the CD8^+^ T cell response following MHV68 infection (**Figure 2**). However, the effects of the type I IFN response on the CD8^+^ T cell response are probably mediated by non-T cells, because T cell-specific abrogation of type I IFN signaling shows no effect (Jennings et al., 2014). Hence, the type I IFN response may control the MHV68-specific T cell response in an indirect manner by regulating the extent of MHV68 replication.

Similarly, it has recently been shown that the type I IFN response contributes significantly to the control of MHV68 latency (**Figure 2**). Adoptive transfer of MHV68 latently- splenocytes into *Ifnar1^-/-^* recipient mice led to higher virus propagation and dissemination as well as higher risk of virus reactivation in *Ifnar1^-/-^* mice (Schwerk et al., 2022). Accordingly, type I IFN-deficient mice can only survive low dose MHV68 infection (Barton et al., 2005). During EBV infection, type I IFN responses by pDCs can transiently dampen virus replication and thereby suppress CD8^+^ T cell proliferation (Gujer et al., 2019). But even without type I IFN signaling, T cells can infiltrate the brain and spinal cord of MHV68-infected *Ifnar1^-/-^* mice, revealing the impact of IFN-independent inflammatory pathways for T cell migration (Márquez et al., 2022).

The contribution of central downstream signaling factors of the type I IFN response has also been described. For example, interferon regulatory factor 1 (IRF1) is an antiviral transcription factor and tumor suppressor (Tamura et al., 2008; Panda et al., 2019). IRF1 expression is robustly induced in epithelial cells in response to IFNβ, and IRF1-deficient mice are more susceptible to viral infections (Novatt et al., 2016). Expression of IRF1 leads to selective attenuation of the MHV68-driven germinal center reaction in a global and T cell intrinsic manner, hence restricting the expansion of the latent MHV68 reservoir (Jondle et al., 2021). IRF1 deficiency leads to an increase and expansion of IL17A expressing CD4^+^ T cells as well as follicular T helper cells which are critical for the initiation of the germinal center reaction, indicating a role for IRF1 in suppressing subpopulations of CD4^+^ T cells that support chronic MHV68 infection. Accordingly, IRF1 deficiency resulted in an increase in the frequency of germinal center B cells, Tfh cells as well as the latent MHV68 reservoir in the spleen and peritoneal cavity of mice following MHV68 infection (Jondle et al., 2021).

Together, these recent studies highlight that the gammaherpesvirus-triggered type I IFN response influences the T cell response on different levels. Firstly, type I IFN acts directly on antigen-presenting cells and on responding T cells themselves. Secondly, type I IFN activity helps to minimize gammaherpesvirus spread and thereby keeps the viral load under control (**Figure 2**). In this way, a reduced gammaherpesvirus load in the host leads to reduced overall lytic gene expression, thereby limiting the available amount of antigen for T cell stimulation. This tuning of viral antigen availability by type I IFN is therefore a major factor for T cell proliferation and activation.

## The NK cell-mediated response during EBV and MHV68 infection

Natural killer cells are major players in the host response to viral infection. Known as a member of innate lymphoid cells (ILC) with cytotoxic properties, they were first identified by their ability to lyse tumor cells *in vitro* (Kiessling et al., 1975; Oldham, 1983). NK cells derive from common lymphocyte progenitors and reside in peripheral blood, lymphoid organs, and various other tissues (Carrega and Ferlazzo, 2012). Although they have similar functions as cytotoxic CD8^+^ T cells, NK cells do not need prior antigen exposure and priming. They express germline-encoded receptors to sense their environment, including inhibitory receptors, e.g., Killer Ig-Like Receptors (KIR) in humans and members of the Ly49 family in mice, and activating receptors, e.g., NKG2D, DNAX accessory molecule-1 (DNAM-1) in mice and humans and Natural cytotoxicity receptors (NCRs) (e.g. NKp46) in mice (Nabekura et al., 2014; Abel et al., 2018). NK cells sense inflammatory signals and become activated and expanded through various pathways: *via* their cytokine receptors (IFNAR1, IL-12R, IL-15R, and IL-18R) (Vivier et al., 2008), *via* their Fc gamma RIII (CD16) receptor (Lee et al., 2015), and by activating receptors expressed by the majority of NK cells (Bottino et al., 2005).

During viral infection, NK cells are alerted by cytokines, such as type I IFN, IL-12, or IL-18 (Hammer et al., 2018). After activation, NK cells exert their role *via* two effector functions, first by contact-dependent cytotoxicity (Krzewski and Strominger, 2008). This function includes recognizing, contacting, and establishing an immunological synapse with the target cell and inducing apoptosis by ligation of FasL and TRAIL ligands (Khosravi-Far and Esposti, 2004), or by degranulation of Granzyme B and Perforins (Bhat and Watzl, 2007). As a second effector function, NK cells also secrete a range of proinflammatory cytokines and chemokines, including IFNγ, TNF, and Granulocyte-macrophage colony-stimulating factor, which further activate other immune cells (Fauriat et al., 2010; Vivier et al., 2011).

NK cells probably play a role during gammaherpesvirus infection (Münz, 2021). Accordingly, to counteract this arm of the immune response, human gammaherpesviruses have developed various strategies to evade NK cell-mediated immune responses, mostly by suppressing the signaling of activating receptors and triggering the signaling of inhibitory receptors (Münz, 2021). Multiple observations support the theory that NK cells contribute to the cellular immune response following human gammaherpesvirus infections. For example, EBV-specific CD8^+^ T cells and human NK cells expand during infectious mononucleosis (IM) (Chijioke et al., 2016). Also, genetic polymorphisms of the NKG2D receptor gene axis have been associated with susceptibility to develop EBV-induced nasopharyngeal carcinoma (Viet et al., 2021). Additionally, decreased frequency of cytotoxic NK cells (CD56^dim^ CD16^+^) causes impairment in antibody-dependent NK cell cytotoxicity in patients with EBV^+^ classical Hodgkin lymphoma (Pánisová et al., 2022).

Interestingly, increased activation of the PI3K/Akt pathway and increased levels of STAT1 were observed in NK cells from patients with chronically active EBV infection (Howe et al., 2020). This indicates that NK cells are continuously activated by lytic EBV infection and contribute to the innate immune response against EBV (**Figure 2**). In addition, IFNβ treatment increases the cytotoxicity of NK cells against nasopharyngeal carcinoma cells *in vitro* in a TNF-related apoptosis-inducing ligand (TRAIL)-dependent manner as TRAIL expression levels on the cell surface of NK cells increased following IFNβ treatment in patients (Makowska et al., 2019).

Only few studies on the role of NK cells in the context of MHV68 infection are published yet, and the mechanisms of MHV68-mediated evasion of the NK cell response are not well understood. Although NK cells are activated, expand, and get recruited to the site of infection following MHV68 infection in C57BL/6 mice, they do not significantly contribute to the control of MHV68 acute or latent infection (**Figure 2**) (Usherwood et al., 2005; Thomson et al., 2008). Depletion of NK cells does not lead to significantly higher viral loads in the lungs compared to control mice following intranasal infection. Additionally, NK cells do not seem to play an important role for the development of the adaptive immune response during MHV68 infection, in particular for the expansion of virus-specific CD8^+^ T cells (Usherwood et al., 2005; Thomson et al., 2008). However, another study showed that after subcutaneous footpad MHV68 infection, NK cells restrict the lytic infection of sub-capsular (SCS) macrophages in the infected lymph nodes, suggesting that NK cells may contribute to the immune response in a tissue-specific manner (Lawler et al., 2016). In another study, the authors proposed a CD4^+^ T cell-NK cell axis that is contributing to the control of MHV68 infection in the lungs (Lawler and Stevenson, 2020). In this case, primed virus-specific CD4^+^ T cells migrate to the lungs and drive the activation of local antigen-presenting cells (APC) *via* IFN-γ secretion. The activated APC then recruit and activate NK cells, presumably by secreting IL-12 and IL-18. Subsequently, activated NK cells contribute to the killing of infected cells and suppress further viral replication *via* IFN-γ secretion (Lawler and Stevenson, 2020). Regarding NK cell-mediated contact-dependent cytotoxicity, it was suggested that MHV68-infected cells circumvent this by up-regulating the inhibitory receptor CEACAM1 on the surface of infected cells (Adler et al., 2009; Adler et al., 2014). However, this needs further investigation. Taken together, there is clear evidence in both humans and mice that NK cells are activated following gammaherpesvirus infection. Nonetheless, a dominant role of NK cells in the control of gammaherpesvirus infection has not been found, indicating that the NK cell immune response alone is not strong enough to control gammaherpesvirus infections.

## Mechanisms of T cell-mediated control of EBV and MHV68 infection

The conventional thymus-derived T cells express alpha and beta T cell receptor chains that allow them to specifically bind to short peptides presented in the context of either MHC class I or II. Most mammalian cells express MHC class I to present peptides to CD8-expressing T cells, and CD4-expressing T cells can be activated by peptide recognition by MHC class II expressed on the surface of antigen-presenting cells (Chopp et al., 2023).

CD8^+^ T cells play a crucial role in controlling different phases of MHV68 infection including acute and latent infection as well as reactivation *via* secretion of perforin, granzymes and IFNγ (Topham et al., 2001; Tibbetts et al., 2002; Loh et al., 2004). Likewise, CD8^+^ T cells play a central role in controlling EBV primary infection (Taylor et al., 2015). However, both MHV68 and EBV can establish latency in the host, irrespective of the very strong cellular immune response (Torti and Oxenius, 2012).

### Gammaherpesvirus immune evasion: The role of CD8^+^ T cells and surface MHC class I expression

How MHV68 and EBV evade the MHC class I-restricted CD8^+^ T cell response remains an important question for both immunological and virological studies. Gammaherpesvirus proteins like the K3 protein mediate MHC class I downregulation in KSHV (Brulois et al., 2014) and MHV68 (Stevenson et al., 2002). Furthermore, gammaherpesvirus genome maintenance proteins (GMP) have recently been established to play a role in both latency maintenance and evasion of CD8^+^ T cell immunity (Sorel and Dewals, 2019), following infection with both MHV68 and EBV (Bennett et al., 2005; Ressing et al., 2008). Additionally, the presence of gammaherpesvirus-infected B cells in the thymus could alter T cell development in this primary lymphoid organ, and thereby cause evasion of viral epitopes by depletion of virus-specific CD8^+^ T cells even before such T cells could be activated in lymph nodes that drain gammaherpesvirus-infected tissues (Yamano et al., 2019). To treat latently infected patients, a specific reduction of gammaherpesvirus-mediated MHC class I immune evasion may allow more efficient CD8^+^ T cell immunity. This concept was recently tested in KSHV-infected human umbilical vein endothelial cells *in vitro*, where treatment with CDK4/6 inhibitors was shown to counteract KSHV-triggered MHC class I down-modulation (Wu et al., 2022). Such direct inhibition of viral immune evasion could be beneficial in the context of adoptive CD8^+^ T cell therapy of chronic gammaherpesvirus-infected patients to allow more complete recognition and killing of infected target cells.

### The contribution of CD4^+^ helper vs. CD8^+^ T cells during gammaherpesvirus infection

CD4^+^ T cells also play an important role in immune-cell mediated control of gammaherpesvirus infection. Heterogeneous clones of CD4-expressing T helper cells are generated during MHV68 infection (Hu et al., 2015). It is proposed that CD4^+^ T cell activation during MHV68 infection is mediated by uninfected myeloid cells that present MHV68-derived peptides loaded on MHC class II (Lawler and Stevenson, 2020). This indirect cellular control of herpesvirus infection has been previously shown for MCMV infection models (Lueder et al., 2018) and following different routes of MCMV infection (Xie et al., 2022). By recognition of different gammaherpesvirus-infected cell types, CD4^+^ and CD8^+^ T cells can cooperate to control the infection (Tan et al., 2017). However, further insights into the regulation of CD4^+^ and CD8^+^ T cell responses are needed to allow a better understanding of infection control and host damage limitation during persistent infection.

Direct contact-dependent killing of MHV68- or EBV-infected cells is an important mechanism how the adaptive immune system controls the viral spread in the host (Ressing et al., 2015). In light of this mechanism, adoptive T cell therapy in EBV-infected patients aims to selectively kill EBV-infected cells (Lammoglia Cobo et al., 2022). In ongoing trials, EBV-specific T cells are injected with the purpose to reduce numbers of EBV-infected B cells and thus lower overall disease severity. It is well established that T cells recognize many different gammaherpesvirus epitopes during primary infection and latency (Gredmark-Russ et al., 2008; Freeman et al., 2010). However, it remains unclear (1) which of these epitopes are presented *in vivo* on the different types of MHV68- and EBV-infected cells, and (2) when they are presented during infection. Strong T cell-mediated responses might be triggered against cross-presented epitopes that may be not present at high levels on the actual target cells. i.e. latently infected B cells. Adding additional complexity, the cell type- and tissue-specific innate sensing and resistance mechanisms can determine which cells are productively infected by MHV68 and EBV (Fujiwara and Nakamura, 2020). In this context, it is important to keep in mind that T cell-mediated responses likely differ between control of acute virus infection versus chronic viral infection (Sarawar et al., 2020). This distinction may depend on the different inflammatory milieu during acute and chronic phases of gammaherpesvirus infection, and further studies are necessary to gain knowledge about the infected cell types, triggered innate immune mechanisms, and different MHC class I and class II T cell epitopes presented *in vivo*.

### Gammaherpesvirus control by T cells: Role of T cell activation and costimulation

Costimulatory signals are important in shaping the magnitude and phenotype of virus-specific T cells. In the case of EBV, innate immunity and downstream inflammation can affect T cell responses by shaping signaling in antigen-presenting cells (**Figure 2**). For example, CD27 costimulatory signals from antigen-presenting B cells are needed for effective EBV-specific CD8^+^ T cell immune responses (Deng et al., 2021). Antigen-presenting cells can either stimulate T cell responses (costimulatory pathways) or inhibit T cell responses (coinhibitory pathways), a phenomenon with important roles in e.g. autoimmunity or transplantation (Kean et al., 2017). In the context of gammaherpesvirus-mediated diseases, therapeutic interventions that target co-inhibitory PD-1 signaling might have severe consequences for the patients, because PD-1 sends important inhibitory signals to prevent T cells from causing immunopathology (Volk et al., 2021). Taken together, future studies should determine which stimulatory and inhibitory signals are sent out by gammaherpesvirus-infected antigen-presenting cells. At the current stage, how these mechanisms can be optimized to enhance infection control without risking overt immunopathology is unknown.

In chronic viral infection, constant expression of viral antigens often leads to the loss of T cell functions, a phenomenon well known as T cell exhaustion (Kanev and Zehn, 2021). How T cells interact with persistent pathogens like gammaherpesviruses is determined by the amount of T cell exhaustion as a hallmark of chronic infections (Saeidi et al., 2018). Interestingly, in latently gammaherpesvirus-infected patients, the virus-specific T cells remain largely functional (**Figure 2**) (Cannons et al., 2018). In contrast, in patients with EBV-associated lymphoproliferative disorders, EBV-specific T cells show signs of T cell exhaustion (Nakid-Cordero et al., 2021). Also, in immunodeficient transplant patients, high EBV viral load was associated with T cell exhaustion (Macedo et al., 2011). Future studies are needed to better understand how chronic antigen stimulation can lead to T cell exhaustion in the context of latent or chronically active EBV and MHV68 infections.

Taken together, the antiviral effects mediated by gammaherpesvirus-specific T cells are important resistance factors for the host, but even a fully activated T cells response cannot eradicate chronic gammaherpesvirus infection, even in fully immunocompetent patients. Therefore, a better understanding of how gammaherpesviruses evade the antiviral T cell immune response is needed, especially since gammaherpesviruses have evolved multiple ways how to evade the underlying innate immune responses.

## Final remarks

Clear evidence has accumulated over the past decades that gammaherpesviruses are sensed by the immune system, evoke an immune response, and modulate it to avoid their elimination and achieve the establishment of a lifelong chronic infection. Since the innate immune response plays a crucial role in orchestrating and maintaining a functional and efficient adaptive immune response, herpesviruses shape the adaptive response not only directly, but also by their inhibition of the type I IFN and NK cell response. Clearly, more *in vivo* studies are needed to assess the consequences of viral modulation of the type I IFN and NK cell response on the quality of the adaptive immune response, during acute as well as chronic infection. However, this endeavor is not straightforward considering that viral proteins are often multifunctional, with some of them targeting multiple arms of the immune system or regulating viral replication or gene expression. In addition, although an animal model for EBV exists with the murine MHV68 model, not all findings with MHV68 can be transferred to the human gammaherpesviruses and their human host, raising the importance of developing novel model systems. So far, probably only very few of the major players that determine the magnitude of the T cell response against gammaherpesviral infection have been described *in vivo*. Further, future studies will have to disentangle the role of the immune system for antiviral defense as opposed to its role for efficient viral spread and latency establishment. In addition, the protective efficiency of CD4^+^ and CD8^+^ T cells during gammaherpesvirus infection probably differs greatly with the type of virus-infected target cell, as gammaherpesviruses are known to infect both macrophages, epithelial cells and B cells. Thus, systematic studies are needed in the future to show in a quantitative manner which pathways of the innate immune system are most relevant for controlling gammaherpesvirus infection and how an optimal T cell response can be generated.

## Author contributions

All authors contributed to the article and approved the submitted version.
